# Cardiovascular Complications of Prostate Cancer Treatment

**DOI:** 10.3389/fphar.2020.555475

**Published:** 2020-12-22

**Authors:** Michał Wilk, Anna Waśko-Grabowska, Sebastian Szmit

**Affiliations:** ^1^ Department of Clinical Oncology, Centre of Postgraduate Medical Education, European Health Centre, Otwock, Poland; ^2^ Department of Pulmonary Circulation, Thromboembolic Diseases and Cardiology, Centre of Postgraduate Medical Education, European Health Centre, Otwock, Poland

**Keywords:** prostate cancer, treatment, cardiovascular, complications, cardio-oncology

## Abstract

Treatment of prostate cancer (PC) is a rapidly evolving field of pharmacology research. In recent years, numerous novel therapeutics that improve survival and ameliorate disease control have been approved. Currently, the systemic treatment for prostate neoplasm consists of hormonal therapy, chemotherapy, immunotherapy, radiopharmaceuticals, targeted therapy, and supportive agents (e.g., related to bone health). Unfortunately, many of them carry a risk of cardiovascular complications, which occasionally pose a higher mortality threat than cancer itself. This article provides a unique and comprehensive overview of the prevalence and possible mechanisms of cardiovascular toxicities of all PC therapies, including state-of-the-art antineoplastic agents. Additionally, this article summarizes available recommendations regarding screening and prevention of the most common cardiac complications among patients with advanced cancer disease.

## Introduction

Epidemiological studies evaluating all-cause mortality in cancer populations have revealed that almost 45% of men with prostate disease die from other reasons than cancer itself (Brown et al., 1993), and complications of anticancer treatment may be responsible for a meaningful proportion of these deaths (Beyer et al., 2005). These observations led to further investigations on the causes of noncancer mortality among men with prostate cancer (PC). What we currently know is that patients treated for oncological reasons are at 4–6 times higher risk of death from cardiovascular (CV) diseases than the general population (Sturgeon et al., 2019). Furthermore, cardiovascular diseases (CVD) are the most common comorbidities and the second leading cause of mortality among men with PC (Van Hemelrijck et al., 2010; Epstein et al., 2012). This can be partially explained by shared risk factors such as age, alcohol intake, obesity, and cigarette smoking (Rawla, 2019). Nevertheless, other possible causes can also be responsible for such a relationship, for example, iatrogenic reasons. Over the past decades, the treatment of prostate malignancy has been evolving rapidly, and a plethora of new crucial therapeutics have been approved, including hormonal therapy, chemotherapy, immunotherapy, radiopharmaceuticals, targeted therapy, and supportive agents (e.g., related to bone health). Their presence in everyday oncological practice alters the prognosis of thousands of lives, adding substantial benefits in terms of disease control and survival. Nevertheless, despite numerous undeniable advantages, anticancer therapies carry the risk of developing multiple side-effects that may lead to increased morbidity. CV complications are of great concern as they occasionally pose a higher mortality threat than cancer itself. Unfortunately, PC therapies are no exception and possess a significant CV risk. The main goal of cardio-oncology is to obtain knowledge about the prevalence of CV complications and better understand the possible mechanisms responsible for their occurrence. This relatively new medical discipline focuses on alleviating and managing CV diseases of cancer patients, therefore improving their clinical outcomes (Herrmann, 2019).

The aim of this article is to present a comprehensive overview of the prevalence and treatment of cardiovascular toxicities induced by PC therapies with great emphasis on the personalized cardio-oncological approach.

## Cardiovascular Complications of Prostate Cancer Treatment

A summary of CV complications of PC treatment is shown in Table 1.

**TABLE 1:** Summary of cardiovascular complications of agents used in prostate cancer treatment.

Medication	CV complications	Potential mechanism
**ADT**
GnRH antagonist	Ischemic heart disease	Sarcopenic obesity, lipid profiles (increases in total cholesterol, low-density lipoprotein, high-density lipoprotein, and triglycerides), reduced insulin sensitivity and diabetes**,** increased inflammation, atherogenic plaque formation, and plaque destabilization (Scragg et al., 2004; Faris and Smith, 2010; Tzortzis et al., 2017)
GnRH agonist	Hypertension	
First-generation antiandrogens	Heart failure	
	QTc interval prolongation	
	Atrial fibrillation	
**Second-generation androgen receptor blockers**
Enzalutamide	Ischemic heart disease	Delayed rectifier K^+^ current, enhancement of late Na^+^ current, and decrease in NO production in the endothelium (Ikeda et al., 2005; Salem et al., 2019; Zhu and Wu, 2019)
	Hypertension	
	QTc interval prolongation	
Darolutamide	Ischemic heart disease	Unknown
	Heart failure	
Apalutamide	Ischemic heart disease	Unknown
	Hypertension	
**Androgen metabolism inhibitor**
Abiraterone acetate	Ischemic heart disease	Increased mineral corticoid production, reduced androgen synthesis, and fluid retention (Attard et al., 2008)
	Hypertension	
	Atrial fibrillation	
	QTc interval prolongation	
**Chemotherapy**
Docetaxel	Heart failure	Direct cytotoxic effect, oxidative stress, and endothelial dysfunction (Montero et al., 2005; Hung et al., 2015)
	Left ventricular dysfunction	
**Immunotherapy**
Pembrolizumab	Myocarditis	Autoimmune reaction due to T-lymphocytes activation against cardiac tissue cells (Nishimura et al., 2001; Wang et al., 2010; Longoria and Tewari, 2016)
	Pericarditis	
	Conduction diseases	
	Rhythm disturbances	
	Hypertension	
	Heart failure	
	Ischemic heart disease	
Sipuleucel-T	Hypertension	Unknown
	Cerebrovascular events	

Notes: ADT, androgen deprivation therapy; GnRH, gonadotropin-releasing hormone; QTc, corrected; QT, interval; LDL, low-density lipoprotein; HDL, high-density lipoprotein; NO, nitric oxide.

### Hormonal Therapy

PC is a hormone-dependent disease; therefore, therapies targeting the androgen axis are the mainstay of its treatment. The therapeutic armamentarium is continuously being enriched, and currently, it consists of the following:

- Bilateral orchiectomy
- Gonadotropin-releasing hormone (GnRH) agonists: leuprolide, goserelin, and triptorelin
- GnRH antagonists: degarelix
- First-generation antiandrogens: flutamide, bicalutamide, and nilutamide
- Second-generation antiandrogens: androgen receptor (AR) blockers, including enzalutamide, apalutamide, and darolutamide, and androgen metabolism inhibitor, such as abiraterone acetate

Unfortunately, as with most oncological therapies, the magnitude of the treatment effect is a balance between its on-target effect and its toxicities effect, and nearly all of the hormonal agents mentioned above have the potential to induce CV complications.

#### Androgen Deprivation Therapy

The first form of androgen deprivation therapy (ADT) was a procedure of bilateral orchiectomy performed in the 1940s (Huggins and Hodges, 2002). Nowadays, it encompasses not only surgical but also chemical castration (by means of GnRH antagonist or agonist with or without first-generation antiandrogen). Leuprolide, goserelin, and triptorelin are GnRH agonists, and their administration inhibits the pituitary secretion of gonadotropins: luteinizing hormone and follicle-stimulating hormone. Degarelix is a selective antagonist of GnRH that competitively and reversibly blocks GnRH receptors, which in turn results in a rapid reduction of luteinizing hormone and follicle-stimulating hormone release. Flutamide, bicalutamide, and nilutamide are nonsteroidal, first-generation AR blockers (Harris et al., 2009). Either surgical or pharmacological castration leads to lower serum testosterone level and thus PC tumor shrinkage, prostate-specific antigen level reduction, and improvement in symptoms (Perlmutter and Lepor, 2007).

There are several possible explanations of the negative impact of ADT on the CV system. In preclinical studies on animals, testosterone has a positive effect on QTc interval via increasing K+ channels expression in mice cardiomyocytes (Brouillette et al., 2005) or plays a cardioprotective role against myocardial ischemia (Tsang et al., 2008). In addition, it is also suggested that testosterone can cause arterial vasorelaxation via increasing nitric oxide production in the endothelium (Campelo et al., 2012) or via calcium channel blockage (Scragg et al., 2004). Decreased serum testosterone level due to ADT impairs the balance in the aforementioned physiological processes and thus negatively impacts the CV system. In humans, it is indicated that androgen suppression may additionally induce or exacerbate CV risk factors through alterations in body composition (sarcopenic obesity), lipid profiles (increases in total cholesterol, low-density lipoprotein, high-density lipoprotein, and triglycerides), reduced insulin sensitivity and diabetes (Faris and Smith, 2010). Moreover, ADT may prompt an inflammatory and prothrombotic state that promotes initiation or progression of atherogenic plaque and thus hastens CV system impairment (Tzortzis et al., 2017).

The negative impact of ADT on the CV system is a widely debatable issue. One of the first reports on such a relationship was published in 2006 by Keating et al. (73,196 patients enrolled). The authors reported a 16% increase of the risk of coronary artery disease and sudden cardiac death (*p* = 0.001, *p* = 0.004, resp.) and 11% increase of myocardial infarction (MI) in patients treated with GnRH agonists for locoregional PC (*p* = 0.03) (Keating et al., 2006). Several observational studies also indicated a similar correlation (Saigal et al., 2007; Tsai et al., 2007). In contrast to the above findings, a number of articles did not report such an effect (Efstathiou et al., 2008; Roach et al., 2008; Alibhai et al., 2009; Voog et al., 2016).

In the light of emerging data, the American Heart Association, American Cancer Society, and American Urological Association endorsed by the American Society for Radiation Oncology released a science advisory document that drew attention to the potential negative impact of ADT on the CV system (Levine et al., 2010). In addition, the increased incidence of CV complications among men receiving GnRH agonists for PC was also a subject of a safety-warning notification release from the US Food and Drug Administration, 2010 (FDA drug safety communication: update to ongoing safety review of GnRH agonists and notification to manufacturers of GnRH agonists to add new safety information to labeling regarding increased risk of diabetes and certain cardiovascular diseases, 2010).

In 2011, Nguyen et al. analyzed CV mortality, prostate cancer-specific mortality, and all-cause mortality in terms of ADT use. In this meta-analysis (4,141 patients), the castration treatment was not associated with an increased risk of CV death (relative risk (RR) = 0.91, 95% CI: 0.75–1.10) (Nguyen et al., 2011). A later meta-analysis by Zhao et al. included six observational studies (295,407 patients) of ADT use vs. no ADT. In its conclusion, ADT was associated with both CVD (HR = 1.10, 95% CI: 1.00–1.21) and CV death (HR = 1.17, 95% CI: 1.04–1.32). GnRH agonists with or without antiandrogen significantly led to a higher incidence of CVD and increased CV mortality (HR = 1.19, 95% CI: 1.04–1.36; HR = 1.46, 95% CI: 1.03–2.08, resp.). Orchidectomy or antiandrogen monotherapy use did not result in such correlation (HR = 0.94, 95% CI: 0.85–1.03; HR = 1.15, 95% CI: 0.92–1.43, resp.) (Zhao et al., 2014). Moreover, a significant correlation association between ADT use and the risk of nonfatal CVD (MI or stroke), especially between men receiving GnRH agonists vs. non-ADT population (RR = 1.38, 95% CI: 1.29–1.48) was reported in 2015 by Bosco et al. (2015) (491,258 patients). In 2019, Liang et al. in their meta-analysis (582,171 patients) revealed a significant increase in the occurrence of acute MI and CVD in the ADT group compared with non-ADT group (RR = 1.19, 95% CI: 1.02–1.39, *p* < 0.05; RR = 1.25, 95% CI: 1.11–1.40, *p* < 0.05, resp.). The authors indicated that castration therapy was not associated with a higher prevalence of sudden cardiac death (RR = 1.13, 95% CI: 0.92–1.38, *p* = 0.24). In addition, the duration of ADT was not relevant in terms of acute MI and CVD (RR = 1.31, 95% CI: 0.66–2.63, *p* = 0.44; RR = 1.12, 95% CI: 0.97–1.30, *p* = 0.12, resp.) (Liang et al., 2020).

Another important concern revolves around ADT and venous thromboembolic disease (VTE). According to the Padua Prediction Score, active cancer (defined as local or distant metastases and with chemotherapy or radiation in the previous 6 months) and ongoing hormonal therapy are two of several risk factors for VTE in hospitalized, medically ill patients (Barbar et al., 2010). However, in the Khorana Risk Score, PC is not indicated as a neoplasm with a higher risk of thrombosis (Khorana et al., 2008). Nevertheless, in the population-based study of men diagnosed with nonmetastatic PC (154,611 patients), the use and duration of ADT were associated with the risk of VTE (Ehdaie et al., 2012). In 2018, Guo et al. performed a systematic review and meta-analysis (427,555 patients), which revealed that GnRH agonists monotherapy, GnRH agonist plus antiandrogen, and antiandrogen monotherapy were associated with increased deep vein thrombosis occurrence rate, although statistically significant difference was not observed in the orchidectomy group. Additionally, the authors found that GnRH agonists alone and orchidectomy can increase the incidence of pulmonary embolism (Guo et al., 2018). Clinicians should be 149 also aware that ADT-induced hypogonadism can significantly increase the risk of acquired 150 long QTc syndrome and torsade de pointes (TdP) (Salem et al., 2019).

Available data support the relationship between ADT and CVD (coronary artery disease, MI, and VTE). However, uncertainty remains with regard to sudden cardiac death and cerebrovascular accidents. There are concerns about which type of ADT (pharmacological vs. surgical; GnRH antagonist vs. GnRH agonists with or without oral antiandrogen) carries the highest CV threat. Until now, there is only one prospective trial that tried to answer these questions. It compared GnRH agonist and GnRH antagonist treatment in terms of the occurrence rate of a new CV event in men with preexisting CV comorbidity. The study revealed that patients treated with GnRH antagonist experienced significantly less major cardiovascular and cerebrovascular events than those treated with GnRH agonist (the absolute risk reduction of 18.1%, *p* = 0.032) (Shore et al., 2020). These results are now being tested in the large-scale trial, phase III PRONOUNCE study (NCT02663908).

In addition, the year 2020 brought a new ADT agent, relugolix, which is the first oral GnRH antagonist. In the HERO trial, this drug was superior to leuprolide in terms of not only testosterone suppression but also a safer CV profile (54% lower risk of CV complications in relugolix arm vs. leuprolide cohort) (Margel et al., 2019). Currently, this agent awaits approval decisions.

The issue of CV complications of ADT merits further study and definitely will be an important field of future cardio-oncology research.

### Second-Generation Antiandrogens

#### Androgen Receptor Blockers

Despite prominent responses due to lowering serum testosterone levels by ADT, most patients with metastatic PC will eventually relapse and develop metastatic castration-resistant PC (mCRPC). There are several mechanisms responsible for such progression, for example, point mutations or amplifications in AR genes or changes in androgen biosynthesis (Fujita and Nonomura, 2019). This resistance to ADT led to the development of novel AR blockers that demonstrate efficacy in the CRPC setting. This group is represented by enzalutamide, apalutamide, and darolutamide. Their main mechanism of action is inhibition of interactions between androgens and AR, preventing nuclear translocation of ARs or blocking AR-dependent gene transcription. The end result of these processes is a decrease in PC cell proliferation and tumor size (Rice et al., 2019). On the other hand, AR blockage may develop serious CV complications. In preclinical studies, AR gene knockout mice were reported to have a decreased heart size, reduced heart muscle contraction, and exacerbation of angiotensin II-induced cardiac fibrosis (Ikeda et al., 2005). In *in vitro* studies on cardiomyocyte cell lines, enzalutamide administration was associated with delayed rectifier K^+^ current, longer action potential duration, production of afterdepolarizations, and triggered activity and enhancement of late Na^+^ current, which eventually may lead to QT prolongation (Salem et al., 2019). Although enzalutamide, darolutamide, and apalutamide belong to the same class, the type of CV complications that they may induce is not identical. The reasons for this dissimilarity are unknown; however, it may be partially explained by minor differences in the structure of the aforementioned drugs (Shore, 2017; Ji et al., 2020).

##### Enzalutamide

Enzalutamide received its first FDA approval in 2012 in the mCRPC setting. Currently, it is also a valuable treatment option in nonmetastatic CRPC and metastatic hormone-sensitive PC (mHSPC).

In the AFFIRM study (n = 1,199), which evaluated enzalutamide in men with mCRPC after chemotherapy, the most common cardiac complication was hypertension, which occurred in 6.6% of patients receiving enzalutamide vs. 3.3% in the placebo arm (Scher et al., 2012). Hypertension was also the most common cardiac adverse event in the PREVAIL study (n = 1717, patients with mCRPC before chemotherapy) and ENZAMET study (n = 1,125, patients with mHSPC). It was reported in 13% and 8% of patients from the enzalutamide arm, respectively (Beer et al., 2014; Davis et al., 2019). Additionally, 2% of the patients receiving enzalutamide in the PREVAIL study were reported to experience atrial fibrillation (vs. 1% in the placebo cohort).

In 2018, a study reporting the effectiveness of enzalutamide in non-mCRPC setting (1,401 patients) was published. Major adverse cardiovascular events described as acute MI, cerebrovascular hemorrhage and ischemia, and heart failure (HF) occurred in 5% (n = 48) of patients receiving enzalutamide vs. 3% (n = 13) in the placebo group. Hypertension was diagnosed in 12% of patients receiving study drug vs. 5% in the control arm. The authors highlighted that the risk factors for developing the aforementioned complications were as follows: age >75 years old, history of CV disease, hypertension, diabetes, and dyslipidemia (Hussain et al., 2018).

In light of these emerging data, hypertension has become the most common CV complication of enzalutamide. In recent meta-analysis of seven studies including 7,347 patients, the authors found RR = 2.82, *p* < 0.001, for all-grade and 2.27, *p* < 0.001, for high-grade hypertension in population treated with enzalutamide (Zhu and Wu, 2019).

In terms of other CV complications, a meta-analysis was performed by Moreira et al. They did not find a significant correlation between enzalutamide use and all-grade (RR = 1.06, 95% CI: 0.67–1.65) or grade ≥3 (RR = 0.81, 95% CI: 0.28–2.33) cardiovascular events (i.e., acute MI, hemorrhagic or ischemic cerebrovascular conditions, and HF). Nevertheless, this meta-analysis evaluated only the AFFIRM and PREVAIL studies (2,916 patients in total) (Moreira et al., 2017). The latest data indicate that men receiving enzalutamide and other ADTs are at risk of QT prolongation and torsade de pointes (Salem et al., 2019).

##### Apalutamide

Apalutamide received its first FDA approval in the treatment of patients non-mCRPC in 2018. Among men who were recruited in the SPARTAN trial (n = 1,207), hypertension was the most common CV complication (any grade occurred in 24.8%; grade 3 or 4 in 14.3% of patients). Ischemic heart disease was reported in 4% of patients in the study drug cohort vs. 3% in the placebo arm (Smith et al., 2018). In the TITAN trial (n = 525), the incidence of hypertension and cardiac ischemia during apalutamide treatment was 17.7% and 4.4%, respectively (Chi et al., 2019). In both studies, six deaths linked to acute coronary artery disease occurred in the apalutamide group.

##### Darolutamide

The safety and efficacy of darolutamide were evaluated in the ARAMIS trial (n = 1,509). In the darolutamide cohort, serious adverse events were reported in 25% of participants that resulted in the death of 3.9% of the patients. Fatal CV complications were HF (0.3%), cardiac arrest (0.2%), and pulmonary embolism (0.2%). Additionally, nonfatal ischemic heart disease (4.0 vs. 3.4% on placebo) and HF (2.1% vs. 0.9% on placebo) were reported. No clinically relevant difference between darolutamide and placebo in the incidence of hypertension was reported (Fizazi et al., 2019).

#### Androgen Metabolism Inhibitor: Abiraterone Acetate

Abiraterone acetate is a selective inhibitor of the CYP17A1 (17 alpha-hydroxylase/C17,20 lyase) enzyme. CYP17A1 is responsible for catalyzing the androgen synthesis. This enzyme is present in several tissues, e.g., testes, adrenal glands, and PC tumors. Abiraterone impedes the transformation of 17-hydroxypregnenolone to dehydroepiandrosterone, which in turn results in lowered serum testosterone levels (O’Donnell et al., 2004). In addition, abiraterone acetate decreases serum cortisol level, resulting in physiological stimulation of the hypothalamic-pituitary axis and adrenocorticotropic hormone release (Attard et al., 2012). This adaptive rise of adrenocorticotropic hormone induces accumulation of mineralocorticoids, which eventually causes clinical complications such as fluid retention, hypertension, or hypokalemia (Attard et al., 2008). Prednisone or methylprednisolone is mandatory to counteract mineralocorticoid excess.

CV complications of abiraterone were reported in several pivotal trials. In 2011, in the COU-AA-301 trial (1,195 patients with mCRPC after chemotherapy), there was no significant difference in the incidence of CV complications (grade < 3) during abiraterone treatment vs. placebo (13% vs. 11%, resp., *p* = 0.14). The most common cardiac complications in the experimental cohort were rhythm disturbances, i.e., tachycardia (3%) and atrial fibrillation (2%). There was no significant increase in fatal cardiac events in the abiraterone acetate group (1.1 vs. 1.3% in the placebo group) (de Bono et al., 2011).

In the COU-AA-302 trial (1,088 patients with mCRPC before chemotherapy), CV complications (all grades: ischemic heart disease, MI, supraventricular tachyarrhythmia, ventricular tachyarrhythmia, and HF) occurred in 19% of patients treated with abiraterone plus prednisone vs. 16% in the prednisone + placebo group. Cardiac toxicity tended to appear later than >3 months after the treatment initiation (Ryan et al., 2013). In 2018, as a result of the LATITUDE trial, abiraterone received FDA approval in the treatment of mHSPC. The study compared ADT + abiraterone + prednisone vs. ADT alone (1,199 patients). The evaluated combination significantly increased OS and radiographic progression-free survival. Grade 3 hypertension was reported in 20% of patients (Fizazi et al., 2017). A meta-analysis of prospective randomized clinical trials (5,445 patients) revealed that, during abiraterone acetate treatment, RR of all-grade hypertension was 1.80, 95% CI: 1.47–2.19, *p* < 0.001, and the risk of high-grade hypertension was even higher (RR = 2.11, 95% CI: 1.66–2.68, *p* < 0.001) (Zhu and Wu, 2019). A link between abiraterone acetate 267 theraoy and acquired long QTc syndrome and TdP was also found (Salem et al., 2019).

The use of second-generation antiandrogens significantly increased the incidence of cardiovascular events in patients with metastatic PC (Iacovelli et al., 2018). Therefore, there has been a growing concern about their safety in real-world populations, especially in the elderly or those with CV diseases. A population-based retrospective study from The Surveillance, Epidemiology, and End Results (SEER) database of patients using abiraterone or enzalutamide (n = 3,876) revealed that men with coexisting CV comorbidities have a higher mortality threat compared to individuals without a history of CV disorders (Lu-Yao et al., 2020). However, there is also evidence that appropriate control of CV comorbidities before the treatment initiation may substantially reduce the CV risk of the prescribed therapy (Verzoni et al., 2016). Currently, no real-world data studies on CV complications induced by apalutamide or darolutamide are available.

## Chemotherapy

Until 2004, there was no consensus on standard chemotherapy for mCRPC that improved OS. In 2004, due to the results of the TAX327 and SWOG 9916 trials, docetaxel had become the chemotherapy of choice in men with mCRPC (Petrylak et al., 2004; Tannock et al., 2004). Currently, it is also approved in mHSPC (James et al., 2016; Kyriakopoulos et al., 2018). Several years later, cabazitaxel has become the second chemotherapeutic agent for men with PC. Currently, these two drugs are cornerstones of chemotherapy in clinical practice.

### Docetaxel

Cardiac microtubules may play a role in the regulation of heart muscle contraction or can be involved in the pathogenesis of cardiac hypoxia/ischemia or hypertrophy (Webster, 2002). Docetaxel is an anticancer drug that stimulates tubulin to form into permanent microtubules and inhibits their breakdown, resulting in a significant reduction in the amount of free tubulin. This mechanism is responsible for the anticancer activity of docetaxel; however, this action may also impair the CV system (Montero et al., 2005). In addition to this direct cytotoxic damage to cardiomyocytes, docetaxel may induce oxidative stress and endothelial dysfunction via increased cell apoptosis (Hung et al., 2015). Clinically, this agent is widely used in many solid tumors, e.g., breast, lung, gastric, or head and neck cancer. The use of this agent is burdened with a number of clinical complications, and one of the most severe is cardiotoxicity. The incidence of left ventricular dysfunction and HF associated with this agent ranges between 2.3 and 8%. Docetaxel may also induce or exacerbate MI, with an occurrence rate of approximately 1.7% (Yeh and Bickford, 2009). It is worth emphasizing, however, that the incidence of these complications depends on whether docetaxel is used as monotherapy or as a part of multidrug regimens (which also include other potentially cardiotoxic drugs). In PC, docetaxel is currently used as a single-agent chemotherapy. Nevertheless, clinicians should be aware of possible CV complications during this treatment.

### Cabazitaxel

Cabazitaxel is an anticancer agent that inhibits cell cycle by binding to tubulin, a structural protein of microtubules, therefore stimulating their stabilization, which results in suppression of mitotic and interphase cellular function (Pean et al., 2012). Despite many similarities with docetaxel, e.g., regarding the mechanisms of anticancer action, the CV risk profile of both drugs seems to be different. In the TROPIC trial (755 patients), all-grade CV adverse events were more commonly seen in cabazitaxel (vs. mitoxantrone), of which grade ≥ 3 arrhythmias were observed in six patients (1.6%). In the cabazitaxel group, the incidence of tachycardia was 1.6% (none was grade ≥ 3), and atrial fibrillation was 1.1%. Cases of HF have been reported more frequently with cabazitaxel: the event was reported in two patients (0.5%) and one was fatal. One patient (0.3%) experienced fatal ventricular fibrillation, and two patients (0.5%) experienced cardiac arrest. Nevertheless, none of the above was considered by the investigators to be associated with the use of cabazitaxel. The relatively low occurrence of CV complications can be partially explained by the fact that a history of congestive HF, MI in six months before study inclusion, untreated rhythm disturbances, coronary artery disease, and hypertension were the exclusion criteria for the trial participation (Bono et al., 2010). In the PROSELICA study, which compared cabazitaxel doses of 20 vs. 25 mg/m^2^ in 1,200 individuals with mCRPC whose disease progressed after docetaxel, no CV safety concerns have been raised (Eisenberger et al., 2017). Further research from real-world clinical experience is needed to assess if cabazitaxel carries a significant CV threat.

## Immunotherapy

The role of the immunologic system in the course of cancer has been a hot topic for many years. Nowadays, immunotherapy can be a valuable option for specific groups of PC patients. Two immunotherapeutics are available, sipuleucel-T and pembrolizumab.

Sipuleucel-T is the first cancer “vaccine” that is derived from autologous peripheral blood mononuclear cells, which are stimulated to discern prostatic acid phosphatase, which is highly expressed in PC cells. In its pivotal trial (IMPACT, 512 patients), sipuleucel-T improved OS in men with mCRPC. Hypertension was the only CV complication reported in adverse event summary with an occurrence rate of 7.4% (25 patients) vs. 3% (5 patients) in the placebo arm (Kantoff et al., 2010). In the summary of adverse events induced by sipuleucel-T reported to the FDA, the drug may potentially increase the risk of other CV complications such as MI and cerebrovascular events. The mechanisms of their occurrence remain unknown (Dores et al., 2019).

Pembrolizumab is a representative of immune checkpoint inhibitors. It binds to the programmed cell death protein 1 (PD-1) receptor and blocks the interplay with its ligands PD-L1 and PD-L2. Normally, this interaction results in inhibition of T-lymphocytes and release of cytokines. The use of pembrolizumab prevents the inhibitory signaling, therefore causing immune reactivity and enhancing antitumor immune response (Longoria and Tewari, 2016). In preclinical studies, PD-1–deficient mice were prone to develop myocarditis (Wang et al., 2010) or autoimmune dilated cardiomyopathy (Nishimura et al., 2001). Despite the substantial clinical benefit of pembrolizumab use in various solid tumors, unfortunately, this agent can be prescribed for a minority of patients with PC due to the fact that it is approved in populations with specific gene mutations only (i.e., mismatch repair-deficient or microsatellite instability-high tumors) that occur in approximately 2%–5% of men with PC (Le et al., 2017). CV complications induced by immune checkpoint inhibitors are considered rare; however, their occurrence may be underestimated (Varricchi et al., 2017). Immunotherapy-induced myocarditis is one of the most severe CV complications, with a mortality rate up to 50% (Wang et al., 2018). Other CV side-effects include pericarditis, rhythm disturbances, conduction diseases, and cardiac ischemia (Tajiri and Ieda, 2019). Due to the fact that, currently, there are no dedicated data on cardiac safety of pembrolizumab among men with PC, the exact prevalence of CV complications in his particular population is not known.

## Supportive Treatment

Supportive treatment of PC consists of glucocorticoids and bisphosphonates. Glucocorticoids induce CV risk factors such as obesity, insulin resistance, and glucose intolerance. Corticosteroids induce lipolysis, increase the production of very-low-density lipoprotein, and intensify hepatic accumulation of free fatty acids, which eventually leads to dyslipidemia (Fardet and Fève, 2014). Furthermore, chronic corticosteroid use can directly impact the CV system by hastening the incidence and progression of atheromatous vascular disease and inducing hypertension (Walker, 2007). The risk is higher with a longer duration of steroids intake and is daily dose–dependent (Huscher et al., 2009). Bisphosphonates are widely used in patients with bone metastases. In a recent meta-analysis (9,386 patients), no association with CV complications has been found (Kim et al., 2015). Moreover, these agents present a safe CV profile, even in elderly populations (Kirchmayer et al., 2019).

### Clinical Assessment of Prostate Cancer Patient

Due to the relatively high prevalence of CV complications of PC treatment, we strongly emphasize that every patient should undergo a careful clinical assessment before starting any anticancer procedures. The scope and intensity of such supervision should be based on a variety of factors such as age, type of anticancer drug, patient’s current and prior CV status, other comorbidities, and concomitant medications. Every patient aged 70 + should undergo geriatric screening. The most reliable screening tool according to the International Geriatric Oncology Society guidelines is the G8 scale, which identifies patients who could benefit from comprehensive geriatric assessment and geriatric consultation. It also helps in choosing the appropriate intensity of PC treatment plan as decisions should be made on the basis of health status evaluation and not according to chronological age (Droz et al., 2017). Regular cardio-oncology evaluation is strongly recommended. Current CV comorbidities should be well-controlled and optimally treated. Before the initiation of any anticancer procedures, every patient should be screened for hypertension, dyslipidemia, and prediabetes/diabetes. Electrocardiography and transthoracic echocardiography should also be performed at baseline and before every subsequent treatment line. If CV complications occur, the decision on management should be based on cancer prognosis (early cancer vs. metastatic), life expectancy, and patient’s preferences (Curigliano et al., 2020).

### Summary

The treatment of PC is a rapidly evolving field in oncology. The development of novel anticancer agents and the discovery of new indications for well-known drugs improved outcomes of patients with this disease. Nevertheless, those medications carry the risk of many complications, from which CV is considered one of the most severe. Hormonal and metabolic changes or immune stimulation can exacerbate the imbalance of the CV system and therefore induce hypertension, HF, ischemic heart disease, rhythm disturbances, and venous thromboembolic disease. This awareness that men with PC are prone to developing or aggravation of such comorbidities should encourage the development of comprehensive cardio-oncology programs. Baseline screening and personalized evaluation of the CV system are essential for lowering the risk of excess CV mortality in men with PC.

## Author Contributions

Conception or design of the work: MW. Drafting the article: MW and AW-G. Critical revision of the article: MW, AW-G, and SS. Final approval of the version to be published: MW, AW-G, and SS.

## Funding

We declare no funding for the research.

## Conflict of Interest

The authors declare that the research was conducted in the absence of any commercial or financial relationships that could be construed as a potential conflict of interest.
